# Supplementary material for: Smart Nanofiber Mesh with Locally Sustained Drug Release Enabled Synergistic Combination Therapy for Glioblastoma
Source: Nanomaterials (Basel). 2023 Jan 19;13(3):414. doi: 10.3390/nano13030414 (PMC9919272; doi:10.3390/nano13030414)
Supplement: Supplementary file 1 [file nanomaterials-13-00414-s001.zip › nanomaterials-2159169-supplementary.pdf]

Supplementary Materials

Smart Nanofiber Mesh with Locally Sustained Drug Release Enabled Synergistic Combination Therapy for Glioblastoma

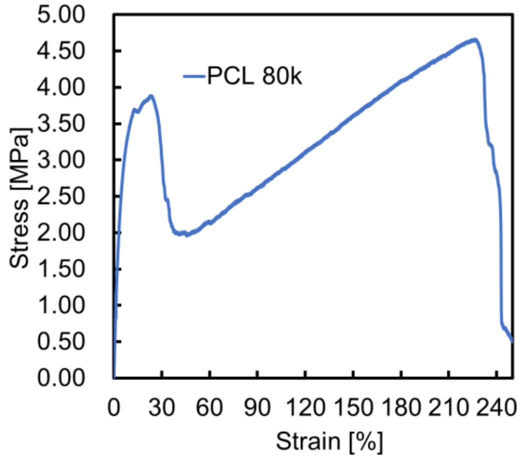

Figure S1. Mechanical property of PCL 80k NFM measured by tensile test.

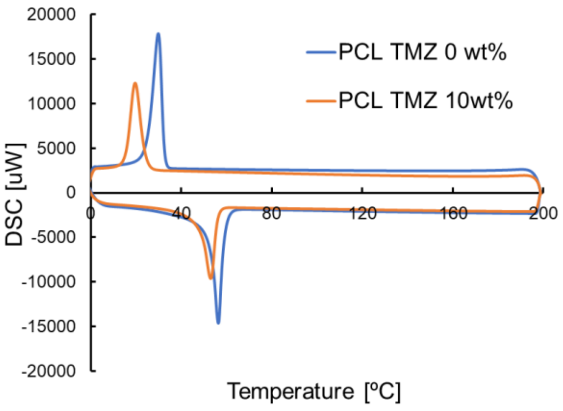

Figure S2. Thermal properties of PCL nanofibers before and after the addition of drugs by measuring DSC.
